# Supplementary material for: Combining spectroscopic and isotopic techniques gives a dynamic view of phosphorus cycling in soil
Source: Nat Commun. 2018 Aug 13;9:3226. doi: 10.1038/s41467-018-05731-2 (PMC6089999; doi:10.1038/s41467-018-05731-2)
Supplement: Supplementary file 1 — Supplementary Information [file 41467_2018_5731_MOESM1_ESM.pdf]

Supplementary Information for

Combining spectroscopic and isotopic techniques to give a dynamic view of phosphorus cycling in soil

Helfenstein et al.

Correspondence to: Julian Helfenstein (julian.helfenstein@usys.ethz.ch)

Supplementary Figures

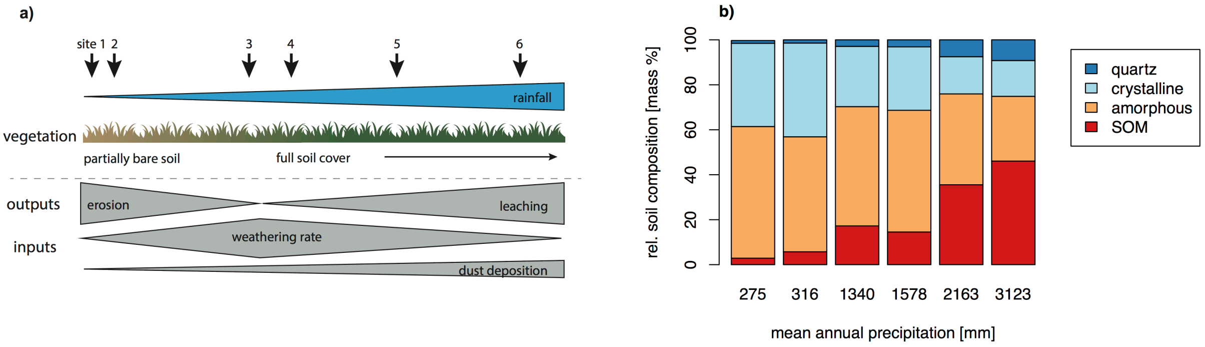

Supplementary Figure 1. Site characteristics along the 150,000-year-old lava flow on Kohala Volcano. All sites were grassland dominated by buffel grass (*Pennisetum ciliare*) or kikuyu grass (*Pennisetum clandestinum*). Panel (a) schematically summarizes climate-driven soil processes based on previous literature on the Kohala gradient; current weathering rates decrease in humid sites because past weathering depleted minerals that could be weatered there. Panel (b) shows relative values of soil components as determined by X-ray diffraction and organic C measurements. Quartz is listed separate from crystalline minerals because it is an indicator of dust deposition. Amorphous = amorphous inorganic; SOM = soil organic matter.

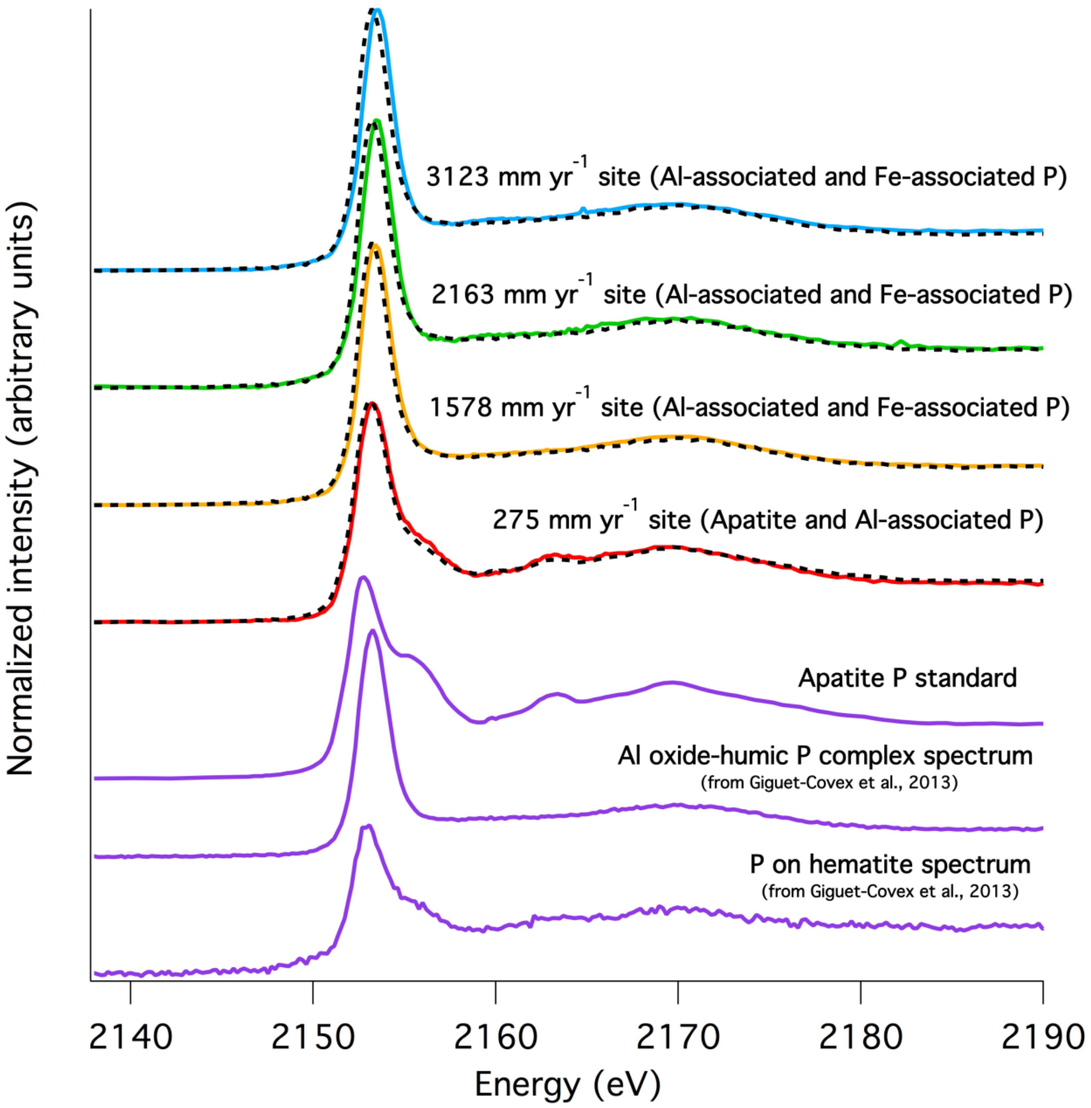

Supplementary Figure 2. P *K*-edge X-ray absorption near edge structure spectra of soil samples and reference spectra. Colored lines show the spectra, dotted lines the fit. Reference spectra were chosen to reproduce key features of the soil spectra, including a pre-edge feature characteristic of P adsorbed on Fe oxides, and post-edge features characteristic of apatite P. All spectra had a significant contribution from Al-associated P, which lacks the distinguishing features of either Fe-associated or apatite P. Soil organic P can be difficult to distinguish from other species using P *K*-edge XANES, so the Al-associated P spectrum was selected to include some features of organic P complexes.

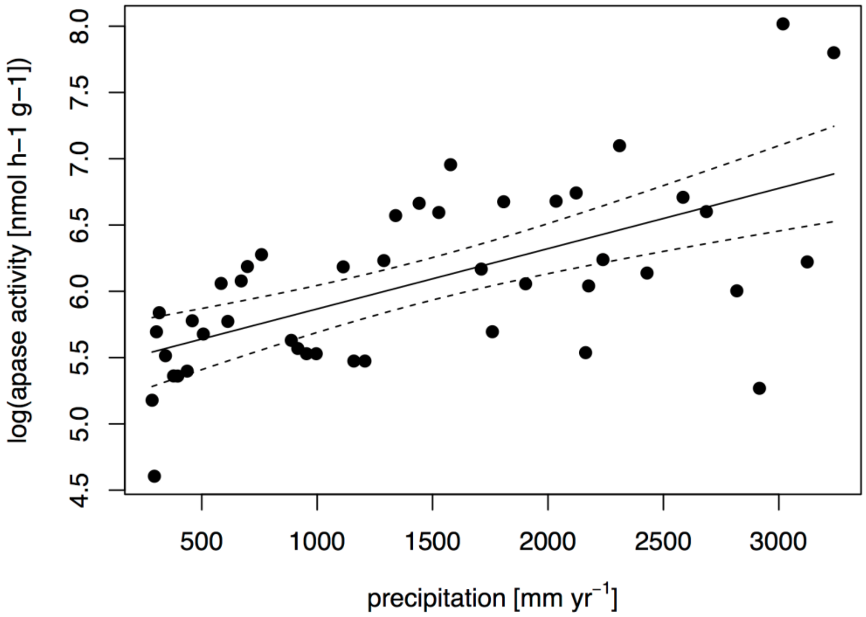

Supplementary Figure 3. Potential acid phosphatase activity along the climatic gradient. Potential acid phosphatase activity was measured on 46 sites along the climatic gradient. The relationship is given by $\log(\text{apase activity}) = 4.55 * 10^{-4}\,(\text{precipitation}) + 5.41$ with an R^2 of 0.38 (F-statistic = 26.4, $p < 0.001$). The dotted lines give the 95% confidence region of the regression curve. Apase = acid phosphatase.

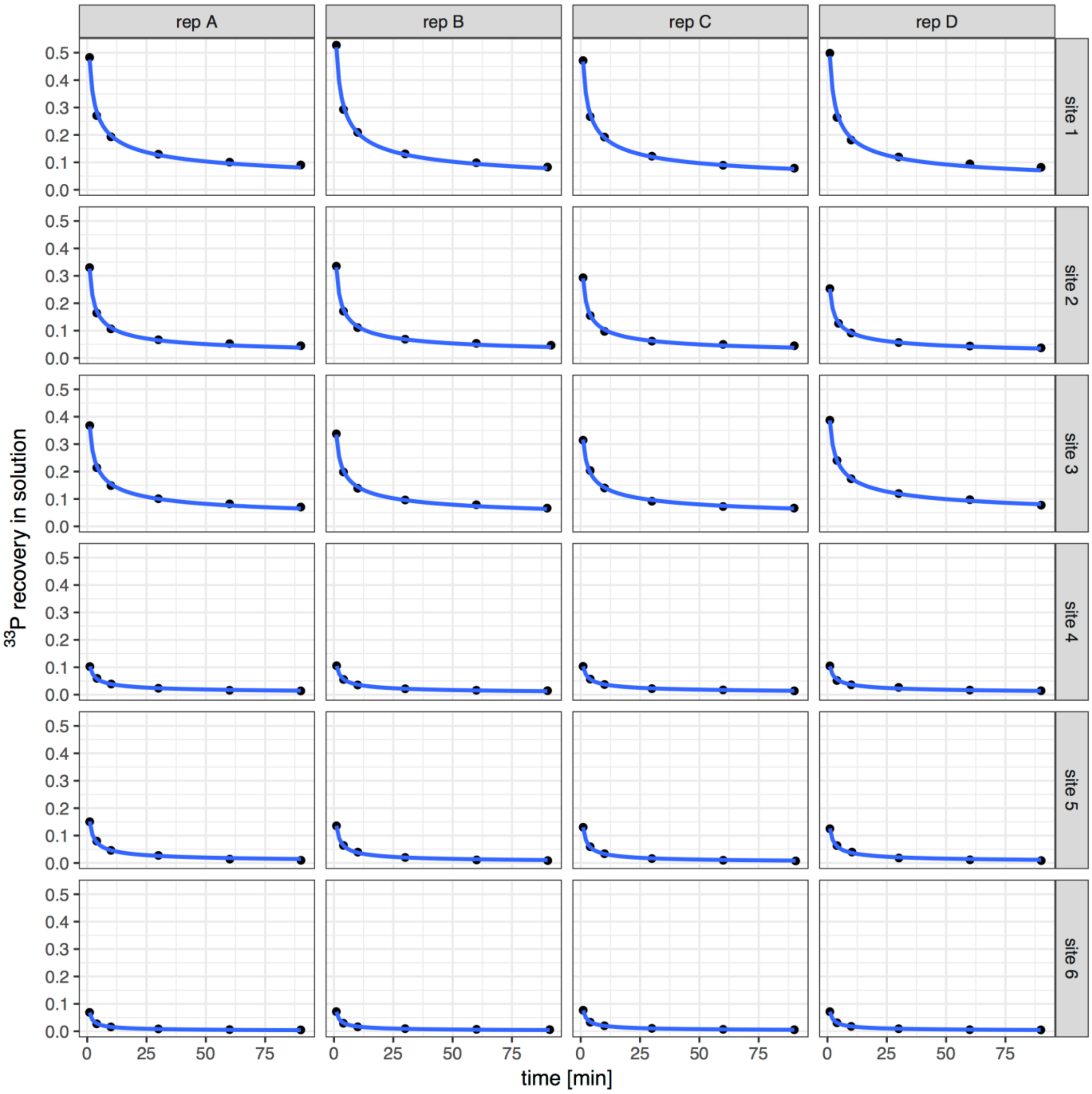

Supplementary Figure 4. Raw data from radioisotope dilution experiments for A horizon soil samples from six sites with four replicates of each experiment. Measured values of ^{33}P recovery were fit using a non-linear model [1] (blue line) to estimate isotope exchange kinetic parameters. The correlation of predicted vs. measured values squared was above 0.995 for all replicates.

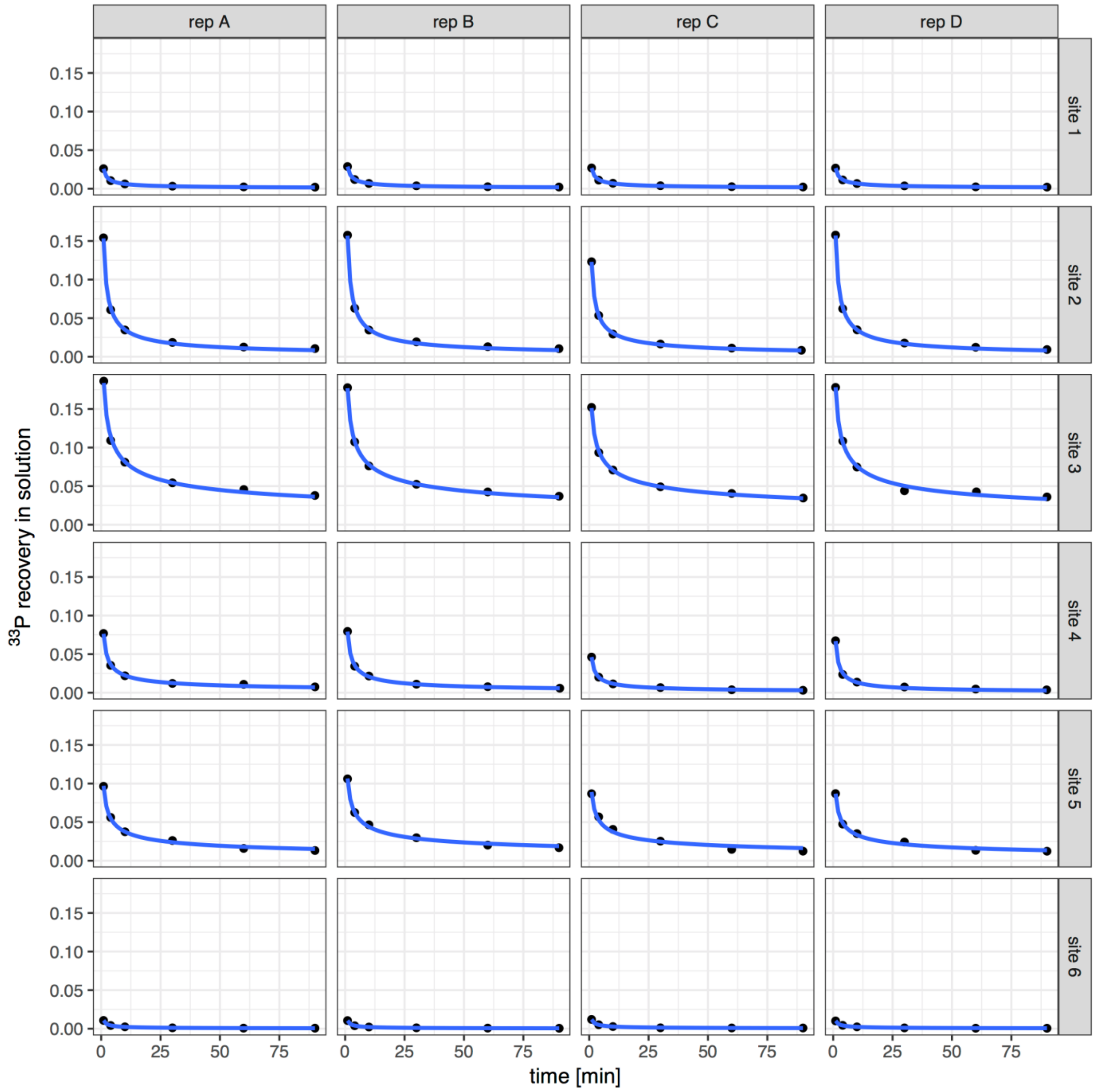

Supplementary Figure 5. Raw data from radioisotope dilution experiments for B horizon soil samples from six sites with four replicates of each experiment. Measured values of ^{33}P recovery were fit using a non-linear model [1] (blue line) to estimate isotope exchange kinetic parameters. The correlation of predicted vs. measured values squared was above 0.984 for all replicates.

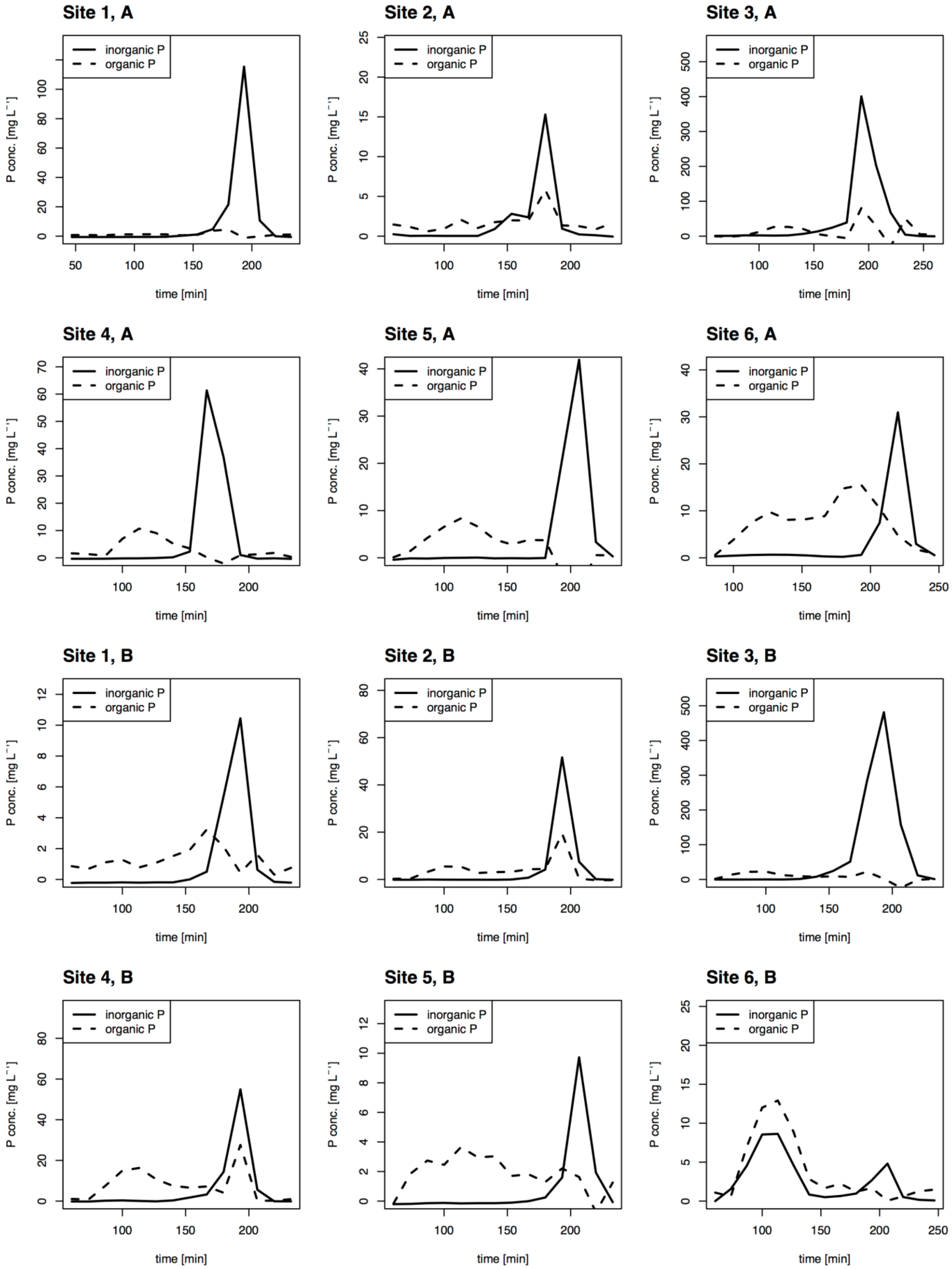

Supplementary Figure 6. Concentrations of inorganic and organic P as a function of elution time from size-exclusion gel chromatography for NaOH-extracts. The first six panels show samples from the A horizon, the following six panels from the B horizon. Organic P was calculated as the difference between total P (measured by ICP-OES) and inorganic P (malachite-reactive P). Peak heights vary based on the amount of NaOH-extract processed. The first peak, usually occurring between 100 and 150 minutes, was considered as the high-molecular weight (HW) fraction, and the second peak, usually occurring at around 200 minutes, was considered as the

low-molecular weight (LW) fraction [2]. HW fractions from sites 1 and 2 were not processed because there was not enough P in these fractions. A clean separation between inorganic and organic P was not possible for site 6, B horizon.

Supplementary Tables

Supplementary Table 1. Extended information on sampling sites.

Site	MAP [mm yr^{-1}]	MAP - PET [mm yr^{-1}]	Elevation [m]	Lat	Long	Sampling depth A horizon	Sampling depth B horizon
1	275	-1487	70	20.113	-155.880	0-6	6-18
2	316	-1499	128	20.116	-155.874	0-8	8-20
3	1340	-328	619	20.148	-155.831	0-15	15-40
4	1578	-77	735	20.151	-155.821	0-30	30-50
5	2163	620	860	20.156	-155.797	0-15	25-50
6	3123	1761	1059	20.135	-155.748	0-10	25-50

Supplementary Table 2. P concentrations in P pools as measured by sequential extraction. Residual P is calculated as total P (determined by X-ray fluorescence) minus the sum of sequentially-extracted pools.

Horizon	Site	Total P [mg kg-^{1}]	Resin-P [mg kg-^{1}]	Microbial P [mg kg^{-1}]	NaOH-Pi [mg kg^{-1}]	NaOH-Po [mg kg^{-1}]	HCl-P [mg kg^{-1}]	Residual P [mg kg^{-1}]
A	1	5229	73.1	14.1	1640	132	1212	2158
	2	5850	67.3	7.3	2111	17	1661	1987
	3	17780	226.1	58.6	9697	1937	4041	1820
	4	6062	87.6	12.3	2606	914	697	1745
	5	5474	47.4	79.1	3277	356	840	874
	6	3641	22.0	159.3	1302	1287	125	746
B	1	4132	1.0	0.3	1790	13	1365	962
	2	4564	29.4	0.0	736	371	1503	1925
	3	20990	384.9	0.0	7034	1491	2152	9929
	4	10590	51.5	6.8	2590	1147	1518	5277
	5	4972	3.4	0.6	669	266	1077	2956
	6	4586	2.8	4.1	1065	607	847	2060

Supplementary Table 3. Phosphorus *K*-edge X-ray absorption near edge structure linear combination fitting results. Fitting was performed using the Athena software package, and fit statistics (*R*-factor, etc.) reported in the table were those computed by the program [3]. Uncertainties computed by the software are shown in parentheses, but the dominant source of uncertainty arises from the choice of standard spectra. Fit results were constrained to fall between 0 and 1, but were not constrained to sum to 1. To compute the proportion of each spectrum, results

were normalized by the raw sum of the proportions. Reference spectra were either collected for this study, or from literature [4].

Site	Apatite P	Al-humic P complex	P on hematite	Raw sum	*R*-factor	χ^2	Reduced χ^2
1	0.632 (0.020)	0.425 (0.014)	0	1.057	0.014	2.68	0.02
2	0	0.716 (0.022)	0.254 (0.040)	0.97	0.023	5.35	0.0399
3	0	0.745 (0.028)	0.226 (0.052)	0.97	0.037	8.97	0.0664
4	0	0.736 (0.033)	0.193 (0.060)	0.929	0.052	12.1	0.0894

Supplementary Table 4. Results from isotope exchange kinetic experiments. Parameters *m* and *n* describe the short and long-term dynamics of P exchange and were calculated using Eq. 1. The ratio r_∞/R was approximated as the proportion of water-extractable P (P_w) to total inorganic P. The mean turnover rate of P in the soil solution, K_m, and the mean flux, F_m, were calculated using the equations from Fardeau et al. [1]. Isotopically exchangeable P, *E(t)*, were calculated for the stated time frames using Eq. 2. Parentheses contain standard errors of the mean.

Hori-zon	Site	P_w [mg P kg^{-1}]	r_∞/R [x 10^{-4}]	*m*	*n*	K_m [min^{-1}]	F_m [mg P kg^{-1} min^{-1}]^a	E_{1min} [mg P kg^{-1}]	E_{1min} - E_{1day} [mg P kg^{-1}]	E_{1day} - $E_{3months}$ [mg P kg^{-1}]	$E_{>3months}$ [mg P kg^{-1}]
A	1	6.72 (±0.016)	23.0 (±0.19)	0.535 (±0.034)	0.437 (±0.018)	1.87 (±0.28)	12.6 (±0.56)	13.8 (±0.64)	261 (±23)	969 (±100)	1682 (±122)
	2	3.64 (±0.033)	9.49 (±0.087)	0.312 (±0.011)	0.472 (±0.0050)	6.03 (±0.59)	22.1 (±2.3)	12.3 (±0.48)	320 (±4.9)	1363 (±22)	2143 (±25)
	3	42.5 (±0.41)	30.4 (±0.29)	0.359 (±0.0089)	0.377 (±0.0050)	6.02 (±0.46)	256 (±20)	121.2 (±3.3)	1518 (±65)	4208 (±164)	8117 (±229)
	4	3.38 (±0.13)	9.97 (±0.37)	0.106 (±0.00089)	0.465 (±0.0043)	58.9 (±2.6)	201 (±14)	31.8 (±1.4)	704 (±25)	1601 (±19)	1054 (±43)
	5	2.47 (±0.037)	5.93 (±0.089)	0.141 (±0.0032)	0.572 (±0.0095)	18.0 (±0.94)	44.9 (±3.0)	17.9 (±0.57)	877 (±51)	2316 (±32)	953 (±83)
	6	0.328 (±0.014)	2.27 (±0.099)	0.0729 (±0.0010)	0.618 (±0.0042)	42.9 (±0.43)	14.2 (±0.75)	4.56 (±0.24)	313 (±17)	858 (±4.8)	273 (±22)
B	1	0.0443 (±0.00088)	0.140 (±0.0028)	0.0271 (±0.00029)	0.607 (±0.0033)	234 (±7.3)	10.4 (±0.37)	1.64 (±0.049)	129 (±5.8)	1120 (±38)	1907 (±43)
	2	0.386 (±0.013)	1.70 (±0.056)	0.153 (±0.0046)	0.649 (±0.0063)	12.4 (±0.97)	4.65 (±0.23)	2.62 (±0.052)	252 (±11)	1325 (±26)	689 (±36)
	3	38.2 (±0.11)	39.9 (±0.11)	0.172 (±0.0042)	0.376 (±0.0050)	46.5 (±6.5)	1770 (±240)	220 (±4.9)	2308 (±38)	3785 (±54)	3257 (±86)
	4	0.966 (±0.063)	2.32 (±0.15)	0.0676 (±0.0038)	0.611 (±0.019)	58.8 (±7.2)	56.9 (±9.1)	15.5 (±1.8)	1035 (±152)	2168 (±69)	941 (±200)
	5	0.726 (±0.023)	4.15 (±0.13)	0.0947 (±0.0022)	0.401 (±0.0048)	151 (±12)	108 (±7.3)	7.66 (±0.18)	124 (±5.2)	447 (±19)	1170 (±24)
	6	0.0302 (±0.0024)	0.158 (±0.013)	0.0109 (±0.00020)	0.652 (±0.0069)	683 (±35)	19.8 (±0.91)	2.81 (±0.25)	281 (±33)	1123 (±28)	507 (±61)

Supplementary Table 5. Measured δ^{18}O values in plants. Metabolic refers to TCA-extractable) and structural refers to NaOH-extractable P in plant leaves. Buffel and kikuyu grass dominated all of the sites, but at site four and six forage soybean and ohia, respectively, were also present.

Site	Plant species	δ^{18}O$_P$ in plant pools		Calculated δ^{18}O$_P$ in equilibrium with plant water		
		metabolic P [‰]	structural P [‰]	δ^{18}O$_{H2O}$ [‰]	ambient T [°C]	δ^{18}O$_P$ [‰]
1	buffel grass (*Pennisetum ciliare*)	34.8	31.8	19.7	25.0	41.6
2	buffel grass (*Pennisetum ciliare*)	33.9	27.9	15.3	25.0	37.3
3	kikuyu grass (*Pennisetum clandestinum*)	24.5	23.1	7.5	20.0	30.3
4	kikuyu grass (*Pennisetum clandestinum*)	24.5	18.1	6.3	20.0	29.1
4	forage soybean *(Neotonia wightii)*	27.0	19.7	3.0	20.0	25.8
5	kikuyu grass (*Pennisetum clandestinum*)	26.1	20.0	4.7	15.0	28.4
6	kikuyu grass (*Pennisetum clandestinum*)	24.0	18.1	2.4	15.0	26.1
6	ohia (*Metrosideros polymorpha*)	23.4	18.0	1.5	15.0	25.1

Supplementary Table 6. Measured and calculated δ^{18}O values of phosphate. δ$^{18}O_P$ of parent material was 10 ‰. The calculated δ$^{18}O_P$ at equilibrium with soil water is at time of sampling. A more conservative equilibrium window to account for longer turnover times was calculated using information from the literature (see methods for details). δ$^{18}O_P$ of P released by specific enzymes was calculated using the δ$^{18}O_P$ of the organic P pool, δ$^{18}O_{H2O}$ and enzyme specific fractionation factors. For sites 1 and 2, where δ$^{18}O_P$ could not be measured in the organic P pool, a δ$^{18}O_P$ of 20 ‰ was used. A.pase and alk.pase stands for acid and alkaline phosphatase, respectively.

**Horizon**	**Site**	**δ$^{18}O_P$ in soil pools**					**Equilibrium at time of sampling**			**Conservative equilibrium**	**Calculated δ$^{18}O_P$ of phosphate released by specific enzymes**		
		resin-P [‰]	microbial P [‰]	NaOH-Po [‰]	NaOH-Pi [‰]	HCl-P [‰]	δ$^{18}O_{H2O}$ [‰]	ambient T [°C]	δ$^{18}O_P$ [‰]	δ$^{18}O_P$ [‰]	a.pase	alk.pase	phytase
A	1	24.2	23.2	-	27.7	17.2	-1.6	25.0	20.4	20.5 – 29.1	12.1	7.1	16.4
	2	23.9	25.4	-	22.5	15.6	2.6	25.0	24.5	20.5 – 29.1	13.1	8.1	17.4
	3	23.1	19.9	20.5	25.4	23.4	-5.6	12.0	18.5	16.4 – 22.6	11.4	6.4	15.7
	4	19.8	20.6	19.8	24.8	22.5	-1.4	12.0	22.8	16.4 – 22.6	12.0	7.0	16.3
	5	22.3	15.7	20.1	21.7	24.2	-3.4	15.0	20.2	18.4 – 22.4	11.7	6.7	16.0
	6	20.5	13.8	20.3	23.9	21.0	-3.0	15.0	20.7	18.4 – 22.4	12.0	7.0	16.2
B	1	17.5	-	-	22.6	10.5	-3.9	20.0	18.9	20.5 – 29.1	11.5	6.5	15.8
	2	22.1	-	-	27.2	11.3	-4.7	20.0	18.1	20.5 – 29.1	11.3	6.3	15.6
	3	19.6	-	20.8	24.5	23.6	-0.8	15.0	22.8	16.4 – 22.6	12.9	7.9	17.1
	4	20.6	20.4	21.2	24.5	26.4	-2.3	15.0	21.4	16.4 – 22.6	12.8	7.8	17.0
	5	30.9	-	19.9	23.6	26.5	-2.5	15.0	21.2	18.4 – 22.4	11.8	6.8	16.0
	6	20.1	-	23.1	23.5	27.3	-3.4	15.0	20.3	18.4 – 22.4	14.0	9.0	18.3

Supplementary Table 7. Proportion of inorganic and organic P in NaOH extract size fractions. HW stands for high-molecular weight and LW for low-molecular weight [5]. These values were used in the mass-balance to calculate $\delta^{18}O_P$ of NaOH-Pi and NaOH-Po.

Horizon	Site	HW		LW	
		% inorganic P	% organic P	% inorganic P	% organic P
A	1	-	-	100.0	0.0
	2	-	-	98.5	1.5
	3	13.4	86.6	90.0	10.0
	4	7.9	92.1	100.0	0.0
	5	6.4	93.6	97.3	2.7
	6	3.2	96.8	88.8	11.2
B	1	-	-	99.9	0.1
	2	1.7	98.3	67.3	32.7
	3	3.2	96.8	87.7	12.3
	4	9.0	91.0	88.6	11.4
	5	0.0	100.0	67.0	33.0
	6	37.3	62.7	-	-

Supplementary References

1 Fardeau, J.-C., Morel, C. & Boniface, R. Phosphate ion transfer from soil to soil solution: kinetic parameters. *Agronomie* **11**, 787-797 (1991).

2 Jarosch, K. A. *et al.* Characterisation of soil organic phosphorus in NaOH-EDTA extracts: A comparison of 31P NMR spectroscopy and enzyme addition assays. *Soil Biology and Biochemistry* **91**, 298-309, doi:10.1016/j.soilbio.2015.09.010 (2015).

3 Ravel, B. & Newville, M. ATHENA, ARTEMIS, HEPHAESTUS: data analysis for X-ray absorption spectroscopy using IFEFFIT. *Journal of Synchrotron Radiation* **12**, 537-541, doi:10.1107/S0909049505012719 (2005).

4 Giguet-Covex, C. *et al.* XANES spectroscopy as a tool to trace phosphorus transformation during soil genesis and mountain ecosystem development from lake sediments. *Geochimica et Cosmochimica Acta* **118**, 129-147, doi:10.1016/j.gca.2013.04.017 (2013).

5 Tamburini, F., Pistocchi, C., Helfenstein, J. & Frossard, E. A method to analyse the isotopic composition of oxygen associated to organic phosphorus in soil and plant material. *European Journal of Soil Science*, doi:10.1111/ejss.12693 (2018).
